# TiO_2_ Nanoparticle-Loaded Poly(NIPA-*co*-NMA) Fiber Web for the Adsorption and Photocatalytic Degradation of 4-Isopropylphenol

**DOI:** 10.3390/gels8020137

**Published:** 2022-02-21

**Authors:** Hideaki Tokuyama, Ryosuke Hamaguchi

**Affiliations:** Department of Chemical Engineering, Tokyo University of Agriculture and Technology, 2-24-16, Naka-cho, Koganei, Tokyo 184-8588, Japan; s183966t@st.go.tuat.ac.jp

**Keywords:** composite fiber web, adsorbent, TiO_2_, photocatalytic degradation, polymer support, *N*-isopropylacrylamide

## Abstract

A TiO_2_ nanoparticle-loaded polymer fiber web was developed as a functional material with the ability to adsorb and photo-catalytically degrade organic pollutants in aquatic media. A linear copolymer of *N*-isopropylacrylamide (primary component) and *N*-methylol acrylamide (poly(NIPA-*co*-NMA)) was prepared, and composite fibers were fabricated by electrospinning a methanol suspension containing the copolymer and commercially available TiO_2_ nanoparticles. The crosslinking of the polymer via the formation of methylene bridges between NMA units was accomplished by heating, and the fiber morphology was analyzed by electron microscopy. 4-Isopropylphenol generated by the degradation of bisphenol A—one of the endocrine-disrupting chemicals—was used as the model organic pollutant. As poly(NIPA) is a thermosensitive polymer that undergoes hydrophilic/hydrophobic transition in water, the temperature-dependence of the adsorption and photocatalytic degradation of 4-isopropylphenol was investigated. The degradation rate was analyzed using a pseudo-first-order kinetic model to obtain the apparent reaction rate constant, *k*_app_. The enhancement of the photocatalytic degradation rate owing to the adsorption of 4-isopropylphenol onto thermosensitive poly(NIPA)-based fibers is discussed in terms of the ratio of the *k*_app_ of the composite fiber to that of unsupported TiO_2_ nanoparticles. Based on the results, an eco-friendly wastewater treatment process involving periodically alternated adsorption and photocatalytic degradation is proposed.

## 1. Introduction

The removal of hazardous organic pollutants from water bodies and industrial waste streams has attracted considerable attention. In this regard, 2,2-Bis(4-hydroxyphenyl)propane—commonly known as bisphenol A (BPA)—is a representative endocrine-disrupting chemical that has been widely used as a raw material of epoxy resins and polycarbonate plastics. The removal of BPA in aquatic media has been accomplished by adsorption [1] and photocatalytic degradation processes using titanium dioxide (TiO_2_) [2,3,4]. In this study, we propose an improved technique using a functional material having the ability to both adsorb and photo-catalytically degrade various organic pollutants in aquatic media.

TiO_2_ is a semiconductor photocatalyst that is chemically stable, non-toxic, abundant, and inexpensive. In photocatalysis, TiO_2_ generates electron/hole pairs under UV light irradiation, and yields reactive oxygen radicals (•OH, •O_2_^−^, and •OOH^−^) in the presence of water and oxygen. These radicals attack and decompose organic compounds. The related photocatalytic degradation pathways are complicated. For example, BPA is converted to various intermediates such as 4-isopropylphenol, formic acid, and acetic acid, and finally to CO_2_ [2].

TiO_2_ nanoparticles have a high specific surface area, which leads to high photocatalytic activity. However, nanoparticles are difficult to handle in practical applications. Therefore, they are loaded on supporting matrices to improve handling and ensure their repeated or continuous utilization in a variety of applications. Various types of composite materials consisting of TiO_2_ nanoparticles and various supporting materials, including carbon materials [5,6] (e.g., activated carbon, carbon nanotubes, and graphene), metal-organic frameworks (MOFs) [7], hydrogels [8,9,10], and polymer nanofiber webs [11,12,13,14,15,16] have been developed. Among these, polymer webs are promising supports because they are flexible and have a larger surface area. Moreover, they can also adsorb pollutants.

Polymer fiber webs are fabricated using an electrospinning method, which is used for producing liquid sprays by the disruptive action of electrostatic forces working on the surface of a liquid at the tip of a capillary nozzle using a high voltage. By evaporating the solvent in sprays composed of a volatile solvent and dissolved polymer, thin polymer fibers with diameters ranging from nanometer to micrometer can be produced. The so-obtained polymer fiber webs have flexibility and a larger surface area, and thus are promising as adsorbents and catalysts. TiO_2_ nanoparticle-loaded polymer nanofibers have been fabricated by electrospinning a suspension of TiO_2_ nanoparticles and a polymer such as polyacrylonitrile [11,12,13], polyvinyl alcohol [14], or poly(*N*-isopropylacrylamide) (poly(NIPA)) [15,16] in an appropriate solvent.

Poly(NIPA) is a typical thermosensitive polymer with a lower critical solution temperature (LCST) of ~33 °C in water [17,18]. It exhibits hydrophilicity and hydrophobicity, respectively, at temperatures lower and higher than the LCST. Using this feature, poly(NIPA) can be used to reversibly adsorb/desorb various organic compounds—such as BPA—in aqueous media by varying the temperature across the LCST to modulate the hydrophobic interaction between the adsorbent and adsorbate [19,20,21,22].

Thus, we herein report the development and characterization of a TiO_2_ nanoparticle-loaded poly(NIPA)-based fiber web as an adsorbent and photocatalyst for organic pollutants in aquatic media. A linear copolymer of NIPA and *N*-methylol acrylamide (NMA), viz., poly(NIPA-*co*-NMA), was used as the support for preparing the electro-spun fibers [23]. After electrospinning, the polymer was cross-linked by heating; NMA has a self-condensable methylol group, and two molecules of NMA form a methylene bridge, i.e., a crosslink when heated. The crosslinking of polymer is necessary to render the fiber web insoluble in aquatic media and also to firmly immobilize the TiO_2_ nanoparticles in the fiber. In this study, we prepared poly(NIPA-*co*-NMA)/TiO_2_ composite fiber web by electrospinning a methanol suspension containing the copolymer and commercially available TiO_2_ nanoparticles. The morphology of the composite fiber was analyzed by scanning electron microscopy (SEM) and transmission electron microscopy (TEM). Furthermore, 4-isopropylphenol, an intermediate with a benzene ring generated by the degradation of BPA, was used as a model of organic pollutants. The temperature-dependence of the adsorption and photocatalytic degradation was also investigated. The decomposition of the benzene ring was easily evaluated using a UV–visible spectrophotometer. The degradation rate was analyzed using a pseudo-first-order kinetic model, and the apparent reaction rate constant, *k*_app_ was obtained. For comparison, the photocatalytic degradation of 4-isopropylphenol was also conducted using unsupported TiO_2_ nanoparticles.

## 2. Materials and Methods

### 2.1. Preparation and Characterization of Poly(NIPA-co-NMA)

The linear copolymer was synthesized by the free radical polymerization of NIPA and NMA using *N*,*N*,*N*′,*N*′-tetramethyl-ethylenediamine (TEMED) as the accelerator and ammonium peroxo-disulfate (APS) as the initiator. The polymerization was conducted in water under a nitrogen gas atmosphere for more than 3 h at 10 °C. The concentrations of NIPA, NMA, TEMED, and APS in the prepolymer solution were 900, 100, 40, and 4 mol/m^3^, respectively. The resulting polymer was washed thoroughly with deionized water to remove the unreacted reagents using a cellulose tubular membrane with a molecular weight cutoff of 13,000. The aqueous solution of the polymer was then dried at 40 °C. The weight-average molecular weight of the polymer was 1.19 × 10^6^, as determined by gel permeation chromatography using polyethylene glycol as the standard. The LCST of the obtained copolymer was determined to be ~40 °C by measuring the transmittance of the aqueous polymer solution at a given temperature using a UV–visible spectrophotometer [18].

### 2.2. Preparation and Characterization of Composite Fibers

**Figure 1:**
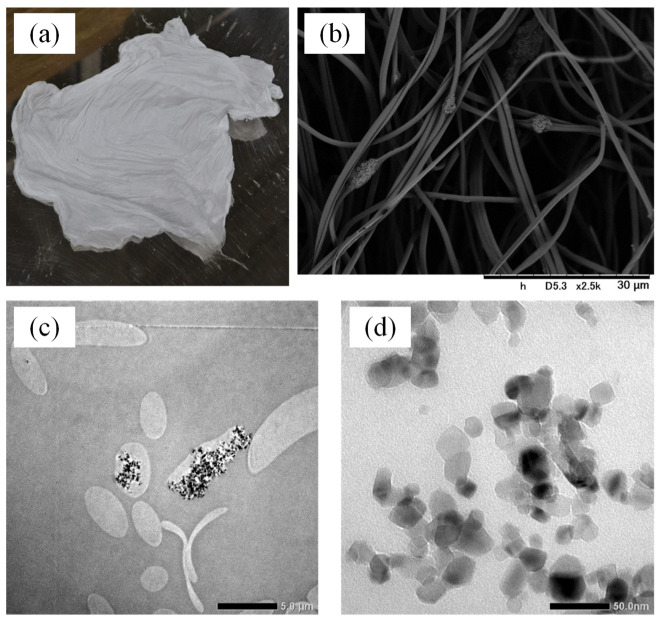
Photographs of TiO_2_ nanoparticle-loaded fiber: (**a**) digital image, (**b**) SEM image (scale bar: 30 μm), (**c**) TEM image (scale bar: 5 μm), and (**d**) TEM image (scale bar: 50 nm).

TiO_2_ nanoparticle-loaded polymer fibers were prepared by electrospinning. TiO_2_ nanoparticles (AEROXIDE^®^ TiO_2_ P25; average diameter: 21 nm) composed of 80% anatase and 20% rutile phases were kindly supplied by NIPPON AEROSIL Co., Ltd. (Tokyo, Japan). A methanol suspension containing NIPA-*co*-NMA polymer (5 wt%) and TiO_2_ nanoparticles (0.5 wt%) was placed in a syringe, fed at a flow rate of 15 cm^3^/h using a syringe pump, and sprayed through a stainless steel nozzle electrode with inner and outer diameters of 0.92 and 1.28 mm, respectively, by applying a voltage of 13 kV using a high-voltage power supply. The spray was collected onto an earthed stainless steel plate placed 15 cm below the nozzle. The fiber web was formed on the plate, and was stripped off it. Figure 1 shows the appearance of the web. The fiber web was subsequently heated at 120 °C for 24 h to enable the crosslinking of the polymer network through the linking of NMA units. The fiber web was finally cut into squares with a side length of 7 cm; the mass of the fiber mat was 0.1 g, and the thickness was ~20 μm.

The TiO_2_ content in the composite fibers was determined by combustion. The dried composite fiber was burned in an electric muffle furnace at 700 °C in an air atmosphere, and the solid TiO_2_ residue was weighed. The determined TiO_2_ content of 0.11 kg-TiO_2_/kg-dry composite on average is comparable to the value (0.091 kg-TiO_2_/kg-dry composite) estimated based on the fabrication conditions. Thus, it was determined that TiO_2_ nanoparticles were successfully loaded on the polymer web.

The structure of the TiO_2_ nanoparticle-loaded fibers was observed by SEM (TM3030, Hitachi High-Tech Corporation, Tokyo, Japan) and TEM (JEM-2100, JEOL Ltd., Tokyo, Japan). As a pre-treatment for SEM, the fiber was coated with gold. As a pre-treatment for TEM, the fiber was embedded in epoxy resin and the resin was sliced using an ultramicrotome. Then, the specimen was placed on a 3 mm diameter Cu grid.

### 2.3. Adsorption and Photocatalytic Degradation of 4-Isopropylphenol

The adsorption experiments were conducted by a batch method. A piece of the TiO_2_ nanoparticle-loaded fiber web was immersed in an aqueous solution (40 cm^3^) of 4-isopropylphenol in a vial. The initial concentration of 4-isopropylphenol was 0.5 mol/m^3^, and it was varied for obtaining the adsorption isotherm. The temperature was maintained constant at 20 °C in an air-conditioned room or a given temperature of >30 °C using an electric griddle. The vial was then placed in the dark for 24 h. The concentration of 4-isopropylphenol in the solution was determined based on its absorbance at 270 nm using a UV–visible spectrophotometer. We confirmed that the adsorption equilibrium was reached after 24 h of incubation. The amount of 4-isopropylphenol adsorbed per unit mass of the dry composite fiber was calculated from the mass balance.

The photocatalytic degradation experiments using the TiO_2_ nanoparticle-loaded fibers were performed after the pre-adsorption process for 24 h, as mentioned above. The photocatalytic degradation of the pollutant was initiated by UV irradiation using a lamp (peak wavelength: 365 nm; 20 W) located at a distance of 8 cm; the solution was aerated. The solution volume was kept constant by adding water when the volume decreased owing to sampling or water evaporation. The concentration of 4-isopropylphenol (*C*) was measured periodically, and the *C*/*C*_0_ ratio was determined (the subscript 0 denotes the initial concentration at time 0). The recyclability of the composite fiber was also assessed. After the degradation had proceeded for 24 h at 50 °C, the fiber was recovered, washed with water, and used for repeated degradation experiments following the pre-adsorption process. For comparison, the photocatalytic degradation experiment was conducted using TiO_2_ nanoparticles (0.01 g) without the pre-adsorption process.

All experiments were performed several times, and the median has been reported.

## 3. Results and Discussion

### 3.1. Morphology of the TiO_2_ Nanoparticle-Loaded Fiber

Figure 1 shows the SEM and TEM images of the TiO_2_ nanoparticle-loaded fibers. In the SEM image, bumps with a few micrometers in diameter are observed on the fibers with ~1 μm diameter. As is clear from the TEM image, TiO_2_ nanoparticles were mainly immobilized in an aggregated state. The particle aggregation should be prevented as it reduces the effective surface area, and thereby the apparent photocatalytic activity. However, we did not optimize the process for achieving the immobilization of the particles in a dispersed state in the current study.

### 3.2. Adsorption Properties

**Figure 2:**
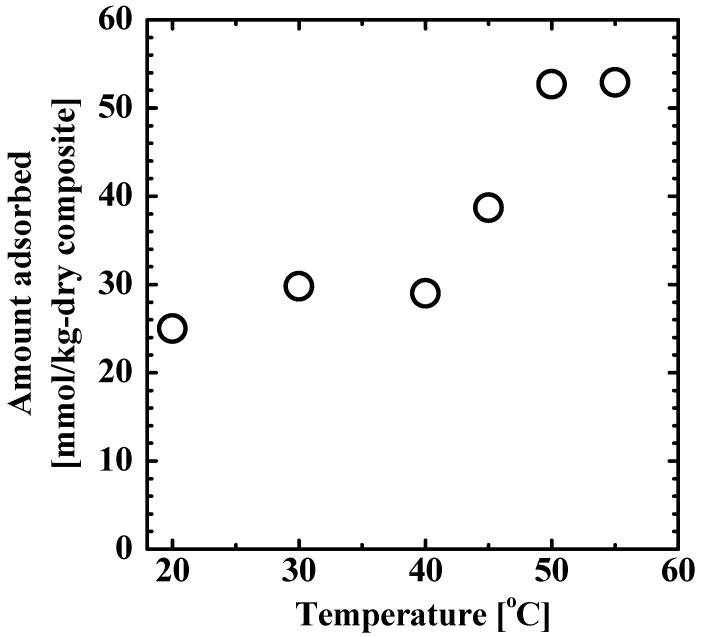
Amount of 4-isopropylphenol adsorbed onto the TiO_2_ nanoparticle-loaded fiber as a function of temperature.

Figure 2 shows the temperature-dependence of the amount of 4-isopropylphenol adsorbed onto the TiO_2_ nanoparticle-loaded poly(NIPA-*co*-NMA) fibers. The adsorbed amount of the pollutant depended on the temperature; it was high at temperatures higher than the LCST and low at temperatures lower than the LCST. The LCST of poly(NIPA-*co*-NMA) was determined to be approximately 40 °C, as mentioned in Section 2.1. The adsorbed amount of 4-isopropylphenol increased with increasing temperature in the range of 40 to 50 °C. The adsorption behavior is mainly governed by hydrophobic interactions. 4-Isopropylphenol can be adsorbed onto the main chain of the polymer below the LCST through hydrophobic interactions and further onto the side chains of NIPA above the LCST.

**Figure 3:**
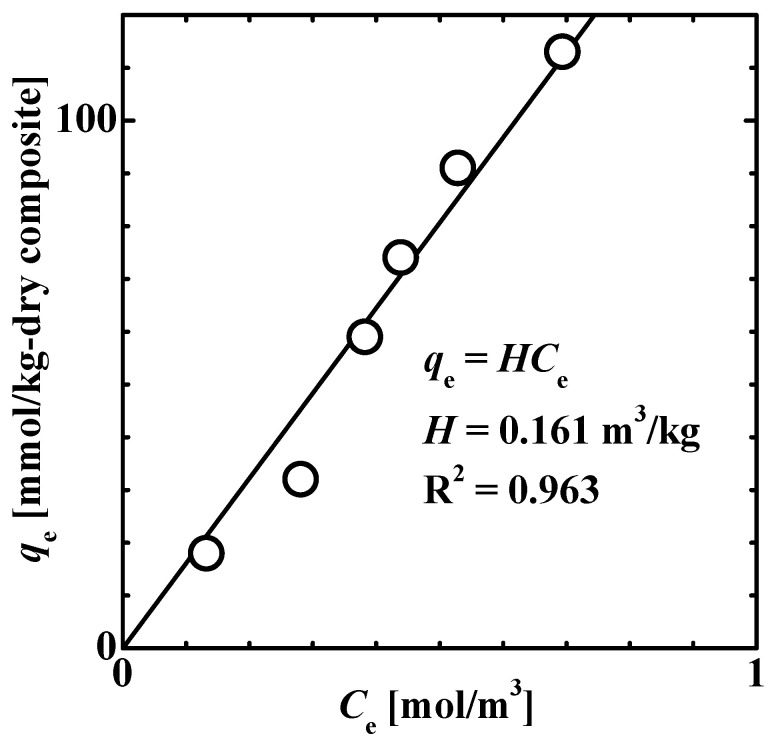
Equilibrium adsorption isotherm showing the relationship between the amount of 4-isopropylphenol adsorbed, *q*_e_ onto the TiO_2_ nanoparticle-loaded fiber and the equilibrium concentration of 4-isopropylphenol, *C*_e_ in the solution at 50 °C. The solid line represents the fit of the data using the Henry-type isotherm function.

Figure 3 shows the equilibrium adsorption isotherm, which reveals the relationship between the adsorbed amount of 4-isopropylphenol, *q*_e_ and the solution concentration, *C*_e_ at 50 °C. The adsorption isotherm could be fitted using the Henry-type isotherm function, expressed as *q*_e_ = *HC*_e_, with *H* = 0.161 m^3^/kg.

### 3.3. Photocatalytic Properties

**Figure 4:**
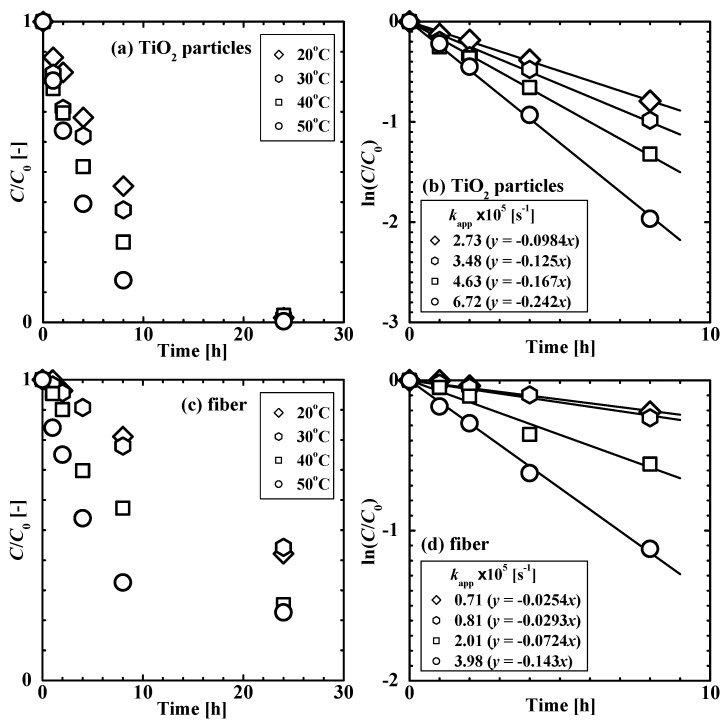
Kinetics of the degradation of 4-isopropylphenol photocatalyzed by (**a**,**b**) TiO_2_ nanoparticles and (**c**,**d**) the TiO_2_ nanoparticle-loaded fiber at various temperatures. (**a**,**c**) Time course of the concentration ratio, *C*/*C*_0_. (**b**,**d**) Analysis of the degradation kinetics using a pseudo-first-order reaction model (Equation (1)).

Figure 4 shows the results of the degradation of 4-isopropylphenol photocatalyzed by TiO_2_ nanoparticles and the TiO_2_ nanoparticle-loaded fiber at various temperatures. The time course of the pollutant concentration ratio, *C*/*C*_0_ indicates that both the TiO_2_ nanoparticles and the TiO_2_ nanoparticle-loaded fiber successfully photocatalyzed the degradation of 4-isopropylphenol.

The kinetics of the reaction in the presence of the two materials were analyzed. The Langmuir–Hinshelwood model is commonly used for the analysis of the photocatalytic degradation of pollutants in the presence of TiO_2_ nanoparticles [3,4,24,25,26]. A sufficiently lower concentration of the organic compound compared to that of the reactive oxygen radicals in this study allowed us to use a pseudo-first-order kinetic model [3,4]. The reaction rate, *r* [mol m^−3^ s^−1^], as a function of the concentration of the organic compound, *C*, and the integral form with *C*_0_ for time *t* = 0 and *C* at a given *t* are as follows:

*r* = d*C*/d*t* = −*k*_app_*C*, ln(*C*/*C*_0_) = −*k*_app_*t* (1)

where *k*_app_ [s^−1^] is the apparent reaction rate constant. The value of *k*_app_ was determined from the slope of the straight line fit of the ln(*C*/*C*_0_) vs. *t* curve obtained for the reaction during the first 8 h, as shown in Figure 4b,d. The *k*_app_ value (3.48 × 10^−5^ s^−1^) for TiO_2_ nanoparticles at 30 °C in this study is comparable to the value reported previously (5.22 × 10^−5^ s^−1^) for the degradation of BPA at 30.5 °C using a similar experimental system [3]. The *k*_app_ value for the case of TiO_2_ nanoparticles is several times greater than that obtained with the TiO_2_ nanoparticle-loaded fiber because the diffusion process of solute through the fiber, i.e., the three-dimensional crosslinked polymer network, can reduce the apparent reaction rate.

**Figure 5:**
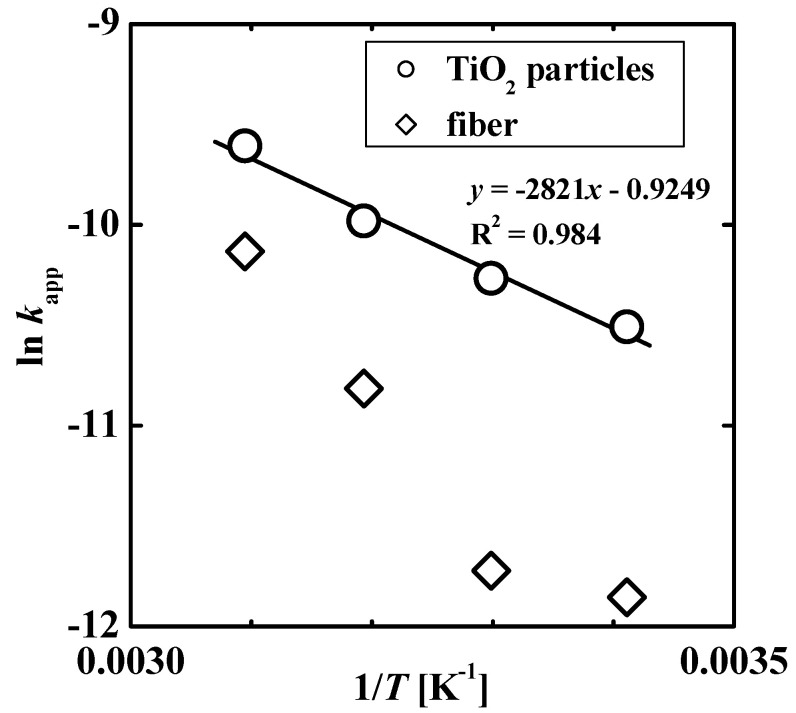
Arrhenius plot for the degradation of 4-isopropylphenol photocatalyzed by TiO_2_ nanoparticles and the TiO_2_ nanoparticle-loaded fiber.

The temperature-dependence of the photocatalytic degradation rate was also analyzed. Figure 5 shows the Arrhenius plot, i.e., the relationship between the logarithm of *k*_app_ and the inverse temperature, *T*. The data for the TiO_2_ nanoparticles exhibited a linear relationship according to the following equation:

*k*_app_ = *A*exp[−*E*_app_/(*RT*)] (2)

where *E*_app_ [J mol^−1^] is the apparent activation energy, *A* [s^−1^] is the frequency factor, and *R* is the gas constant (8.314 J mol^−1^ K^−1^). The values of *E*_app_ and *A* were found to be 23.5 kJ mol^−1^ and 0.397 s^−1^, respectively. The *E*_app_ value is comparable to the value reported (16 kJ mol^−1^) for the degradation of chlorophenol using a similar experimental system [27].

The data for the TiO_2_ nanoparticle-loaded fiber could not be fitted with the Arrhenius equation. This is because the apparent reaction rate is affected by the temperature-dependent adsorption of the solute to the composite fiber, as shown in Figure 2. Further, the effect of the temperature-dependent adsorption on photocatalytic degradation was evaluated using the *k*_app_ values obtained using the two catalysts. The ratio of the *k*_app_ of the composite fiber to that of TiO_2_ nanoparticles was 0.59 at 50 °C and 0.23 at 30 °C. The higher ratio at 50 °C is attributed to the higher amount of solute adsorbed to hydrophobic poly(NIPA-*co*-NMA) in the fiber system. That is, the strong affinity of the hydrophobic pollutant to the fiber can enhance the photocatalytic degradation rate. If TiO_2_ nanoparticles within the fiber are more dispersed (see Figure 1), resulting in an increase in the effective surface area, the *k*_app_ value of the fiber can be comparable to or exceed that of the unsupported TiO_2_ nanoparticle.

**Figure 6:**
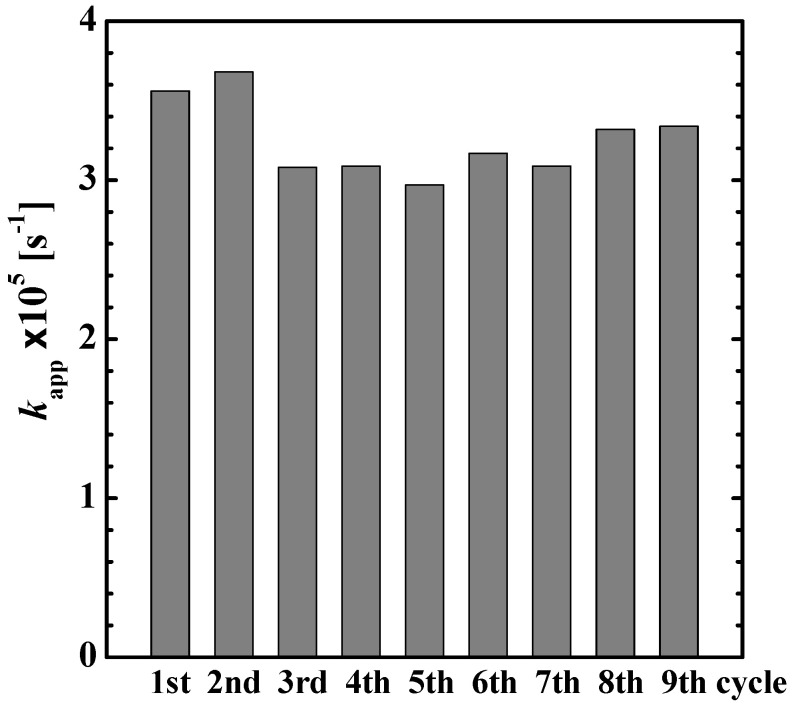
Value of *k*_app_ in the repeated reaction cycles of the degradation of 4-isopropylphenol photocatalyzed by the TiO_2_ nanoparticle-loaded fiber at 50 °C.

We investigated the reusability of the supported photocatalytic system, the TiO_2_ nanoparticle-loaded fiber web in the degradation of 4-isopropylphenol at 50 °C. Figure 6 shows the value of *k*_app_ obtained for each reaction cycle. The comparable values indicate that the fiber could photo-catalyze the degradation adequately and maintain its photocatalytic activity until the 9th cycle. This result indicates that the three-dimensional crosslinked polymer network continued to support the TiO_2_ nanoparticles without suffering degradation. In contrast, the uncross-linked (without heat treatment) poly(NIPA-*co*-NMA) fiber web dissolved in water.

The TiO_2_ nanoparticle-loaded poly(NIPA-*co*-NMA) fiber web can potentially be used in the continuous treatment of wastewater containing a dilute concentration of organic pollutants such as 4-isopropylphenol and BPA. In this process, the wastewater can be passed through a fixed bed column with the fiber web to be purified by the adsorption process. When large amounts of pollutants accumulate on the fiber web, the supply of wastewater can be stopped temporarily, and the photocatalytic degradation can be initiated through UV irradiation, resulting in the regeneration of the fiber web. By performing these adsorption and photocatalytic degradation steps alternately and periodically, wastewater treatment can be performed in an eco-friendly manner.

## 4. Conclusions

A TiO_2_ nanoparticle-loaded poly(NIPA-*co*-NMA) fiber web was fabricated by an electrospinning method using a methanol suspension containing the copolymer and commercially available TiO_2_ nanoparticles, and then heated to crosslink the polymer via methylene bridges derived from NMA units. SEM and TEM images revealed that TiO_2_ nanoparticles were mainly immobilized in an aggregated state within the polymer fibers of ~1 μm diameter. The composite fiber exhibited temperature-dependent adsorption and photocatalytic degradation of 4-isopropylphenol—a byproduct of the degradation of BPA—in aquatic media. The degradation rate was analyzed using a pseudo-first-order kinetic model. The ratio of the apparent reaction rate constant (*k*_app_) of the composite fiber to that of unsupported TiO_2_ nanoparticles indicated that the adsorption of 4-isopropylphenol onto the thermosensitive poly(NIPA)-based fibers can enhance the photocatalytic degradation rate. The composite fiber web can be used repeatedly and is suitable to be used in a fixed bed column for a continuous process of periodically alternated adsorption and photocatalytic degradation cycles.

## Data Availability

Not applicable.
